# Power-law relationship in the long-tailed sections of proton dose distributions

**DOI:** 10.1038/s41598-018-28683-5

**Published:** 2018-07-10

**Authors:** Bo Jiang, Xiaochun Wang, Yang Zhang, Fada Guan, Yupeng Li, Xianliang Wang, Ronald X. Zhu, Xiaodong Zhang

**Affiliations:** 1 Department of Radiation Oncology, Tianjin Medical University Cancer Institute and Hospital, National Clinical Research Center of Cancer, Key Laboratory of Cancer Prevention and Therapy, Tianjin, 300060 China; 2 Department of Radiation Physics, The University of Texas MD Anderson Cancer Center, Houston, Texas 77030 United States; 3 Global Oncology One, Houston, Texas 77054 United States; 4 Department of Radiation Oncology, Sichuan Cancer Hospital & Institute, Chengdu, 610000 China

## Abstract

The halo portion of a proton therapy dose creates a long tail in proton dose distributions, but so far study of this phenomenon has been limited. We used statistical methods and mathematical models to confirm that the long-tailed portion of proton dose distributions exhibits a power-law relationship. By analyzing 299 measured dose profiles, we found that all proton lateral dose distributions had a significant power-law scaling correlation with a high correlation coefficient in the tail. We set up a dual-mechanism model, containing both direct and indirect impact mechanisms. In the direct impact mechanism, the proximal dose deposition is mainly due to the direct impact of a proton on a particle. In the indirect mechanism, the impact of a proton on a given particle is considered in terms of the proton’s impact on a neighboring particle that then impacts the given particle. We found that the indirect impact mechanism led to a tail in the distribution exhibiting a power-law relationship because the probability of the indirect impacts was proportional to the distance; i.e., the longer the distance, the larger the indirect impact probability. Upon analyzing the experimental data, we observed that the power-law exponent increased with proton energy.

## Introduction

Radiation therapy plays an important role in the treatment of cancer; approximately 50% of all cancer patients receive radiation therapy during the course of their illness, and radiation therapy contributes to 40% of curative treatment for cancer^1^. Since Robert R. Wilson recognized the potential advantage of protons for external radiation therapy^2^, proton therapy has been considered a more advanced treatment technique because of its superior physical characteristics. Proton deposit the maximum energy per path length close to the end of range, causing a ‘Bragg’ peak. Owing to the physical characteristics of proton, the maximum dose can be located in the tumor and negligible dose downstream of the Bragg peak. For these reasons, proton radiotherapy has more powerful capabilities to spare the normal tissues and reduce the burden of treatment-related complications compared to photon radiotherapy. Since 1990, more than 150,000 patients have undergone proton therapy^3^, and the number of patients receiving proton therapy has rapidly grown around the world in recent years^4^. Proton therapy clinical research^5–7^ and physical research^8–11^ are currently the most active research areas of radiation therapy.

Protons are charged particles that interact frequently within matter, and these interactions occur through several mechanisms: Coulombic interaction with an atomic electron, Coulombic interaction with an atomic nucleus, nuclear interactions, and Bremsstrahlung^12^. Through these interactions, protons deposit energy on the path of motion, which in proton therapy forms the dose. The measured dose distribution of a proton pencil beam in liquid or solid matter has two parts, the core and the halo^13^. The core and halo overlap, but each has distinct spatial characteristics. The core consists of primary protons that undergo electromagnetic interactions and slow down through multiple collisions with atomic electrons and multiple Coulomb scattering with nuclei. According to the Fermi-Eyges theory^14^, the core’s cross section is Gaussian, typically on the order of a centimeter, and increases with depth. The halo consists of the charged secondary particles and the resulting elastic and nonelastic interactions. The formation of the halo involves very complicated mechanisms, and so far study of halo formation has been limited^9,13,15^. Thus, an alternative definition of the halo might be anything arising from charged particles that falls outside of the Gaussian pattern of the Fermi-Eyges theory^13^. The halo creates a long tail in proton lateral dose distributions. Because most dose distributions have been described on the basis of the Molière theory^16,17^, which leads to Gaussian distribution, the long-tailed portion of the distribution created by the halo has been fitted with empirical models such as two Gaussians with Cauchy-Lorentz function^9^ or three Gaussians^18^. The empirical models have shown partial success in fitting the data, but the mechanism leading to the long-tailed dose distribution remains unclear.

Many long-tailed distributions in a variety of natural and man-made phenomena follow a “power-law” distribution. A power-law distribution in empirical data is a striking feature that has attracted considerable attention^19^. The power-law can relate microcosmic theory to observed macroscopic phenomena. Although power-law distributions have been reported in areas ranging from ecology and molecular biology to finance and the Internet^20–23^, to the best of our knowledge, the power law has not been used to describe the long-tailed proton lateral dose distributions. In the current study, we sought to determine whether the long-tailed proton lateral dose distribution follows the power-law, and if so, whether this phenomenon can be explained by similar mechanisms leading to long-tailed distributions in other research areas. Determining whether the long-tailed proton dose distribution follows the power law will help elucidate the mechanisms of halo formation and thus improve dose calculation.

## Results

**Figure 1:**
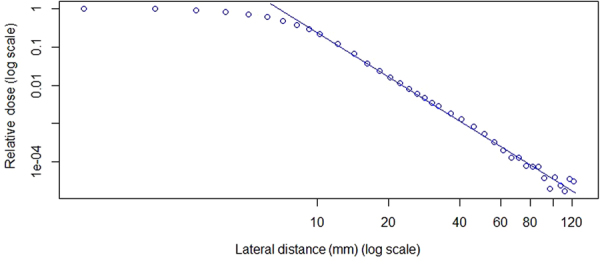
Power-law correlation between lateral distance and dose for 221.8-MeV protons at 5-cm depth, including all measurement points. The power law applies for values in the tail of the profile. Measurement data points are shown as dots and best-fit curves as solid lines.

Figure 1 plots the distance in log scale and lateral dose for 221.8-MeV protons at 5-cm depth in water. The dots show all of the measurement points. We defined the distance from the point with maximum R^2^ when fitting to the power law to the origin as DMO. The straight line in Fig. 1 starting from DMO indicates a strong power-law relationship with R^2^ = 0.994.

**Figure 2:**
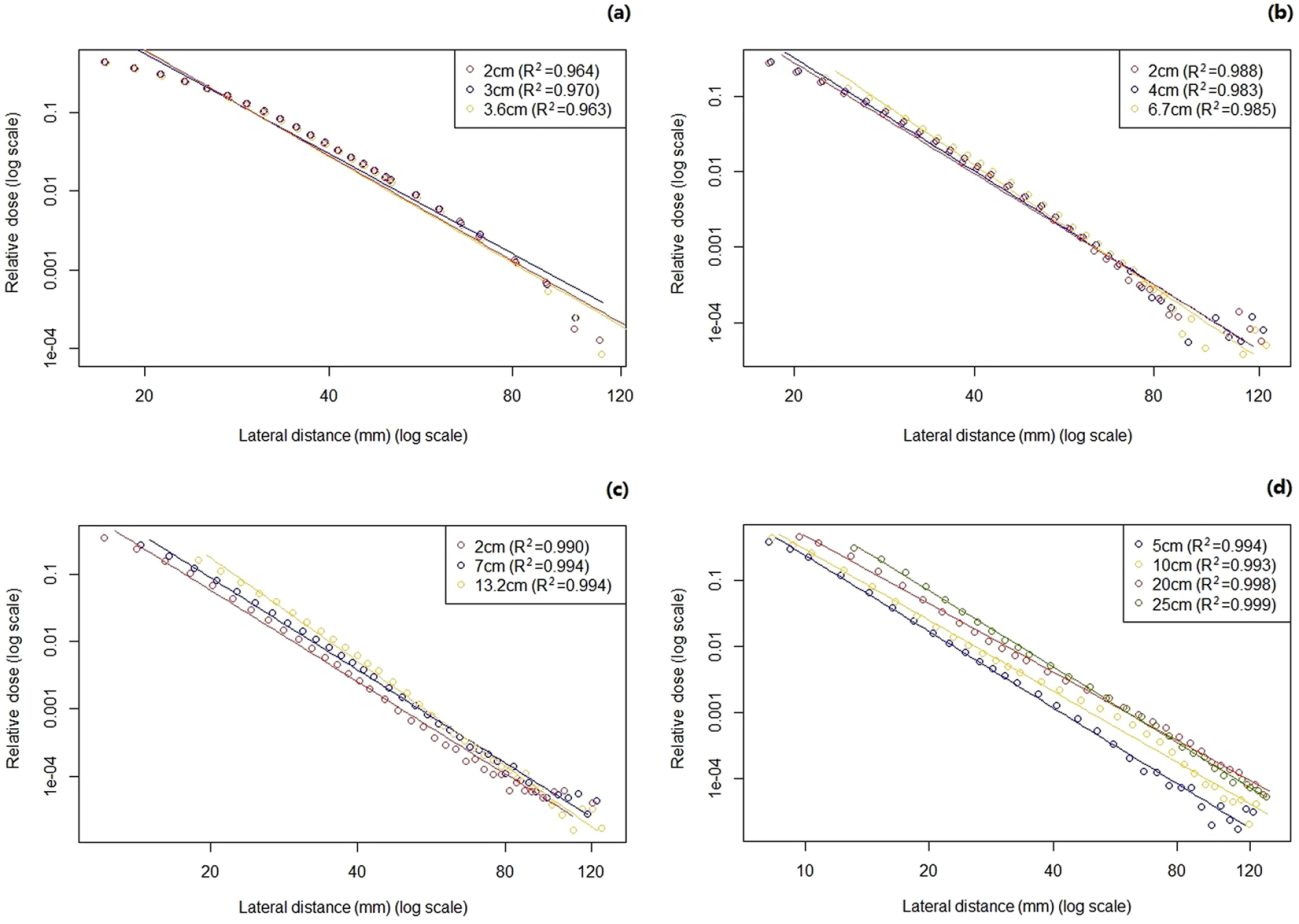
Power-law relationship between the lateral distance and lateral dose for (**a**) 72.5 MeV, (**b**) 102.4 MeV, (**c**) 143.2 MeV, and (**d**) 221.8 MeV, at multiple depths. All proton lateral dose distributions have significant power-law scaling correlations with a high correlation coefficient in the tail. Measurement data points are shown as dots and best-fit curves as solid lines.

Figure 2 shows the results of the power-law correlation between lateral distance and lateral dose for 72.5 MeV, 102.4 MeV, 143.2 MeV, and 221.8 MeV at multiple depths, including the measurement points starting from the maximum R^2^ point. The graph shows that all profiles have significant power-law scaling correlations with a high correlation coefficient.

We calculated the correlation parameters for all profiles (Table 1). All profiles had significant power-law correlations with P < 0.01. The exponent parameter (slope) was −4.41 ± 0.58 with a 95% confidence interval of −4.64 to −4.19. The square of the correlation coefficient was 0.98 ± 0.021, indicating a strong linear correlation between log-transformed dose and distance. The P value and the square of the correlation coefficient indicated that the tail of the profile was strongly consistent with the power-law relationship. These results suggest that the lateral dose decreased 2^4.41^ times each time the lateral distance doubled, staring from the profile data point with the maximum correlation coefficient.

**Table 1:** Parameters of the power-law correlation for lateral distance and dose, using the ordinary least squares line-fitting method.

Energy range (MeV)	No. of profiles	DMO ± SD, mm	α ± SD	95% confidence interval	R^2^ ± SD	P
72.5–79.9	20	25.40 ± 6.64	−4.71 ± 0.73	−5.00 to −4.42	0.98 ± 0.010	0.00
80–89.9	33	24.21 ± 6.49	−4.64 ± 0.68	−4.89 to −4.39	0.99 ± 0.009	0.00
90–99.9	32	19.95 ± 2.77	−4.61 ± 0.58	−4.90 to −4.32	0.98 ± 0.025	0.00
100–109.9	25	20.12 ± 5.09	−4.69 ± 0.57	−4.94 to −4.43	0.99 ± 0.007	0.00
110–119.9	27	19.14 ± 3.26	−4.65 ± 0.33	−4.88 to −4.41	0.99 ± 0.007	0.00
120–129.9	23	16.81 ± 2.08	−4.45 ± 0.41	−4.66 to −4.24	0.99 ± 0.005	0.00
130–139.9	19	15.41 ± 3.79	−4.52 ± 0.40	−4.74 to −4.29	0.98 ± 0.031	0.00
140–149.9	15	15.87 ± 3.10	−4.52 ± 0.39	−4.71 to −4.33	0.99 ± 0.003	0.00
150–159.9	12	12.93 ± 3.36	−4.56 ± 0.34	−4.73 to −4.39	0.99 ± 0.010	0.00
160–169.9	12	12.66 ± 3.82	−4.41 ± 0.44	−4.61 to −4.20	0.98 ± 0.017	0.00
170–179.9	12	10.70 ± 3.15	−4.27 ± 0.36	−4.50 to −4.05	0.98 ± 0.011	0.00
180–189.9	16	10.88 ± 4.77	−4.25 ± 0.58	−4.50 to −4.00	0.96 ± 0.038	0.00
190–199.9	16	9.57 ± 4.28	−3.97 ± 0.44	−4.21 to −3.72	0.96 ± 0.047	0.00
200–209.9	16	9.56 ± 3.30	−3.74 ± 0.30	−3.85 to −3.62	0.99 ± 0.009	0.00
210–221.8	21	9.60 ± 3.19	−3.73 ± 0.34	−3.84 to −3.60	0.99 ± 0.007	0.00
ALL	299	16.63 ± 6.68	−4.41 ± 0.58	−4.64 to −4.19	0.98 ± 0.021	0.00

DMO: distance from the point with maximum R^2^ to the origin; SD: standard deviation; α: slope.

**Figure 3:**
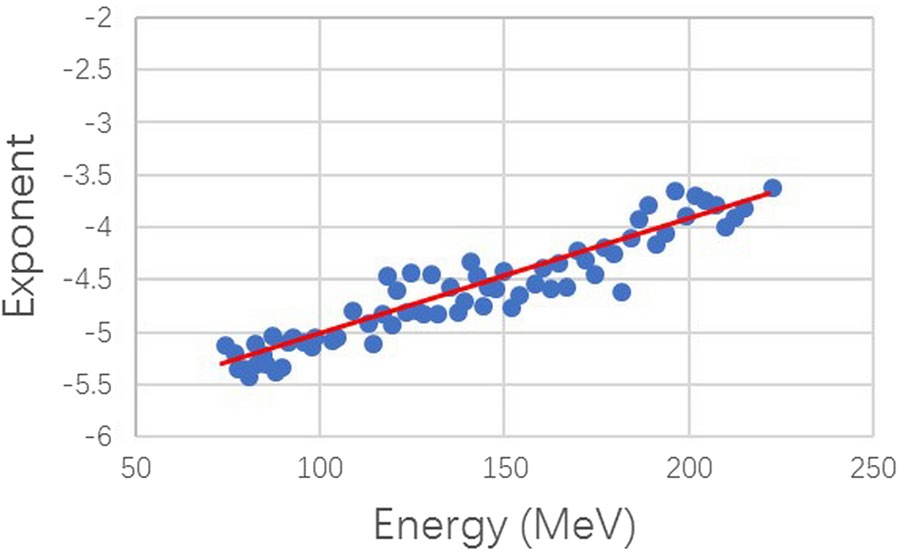
Power-law exponent plotted against energies for profiles. The power-law exponent increases with energy.

**Figure 4:**
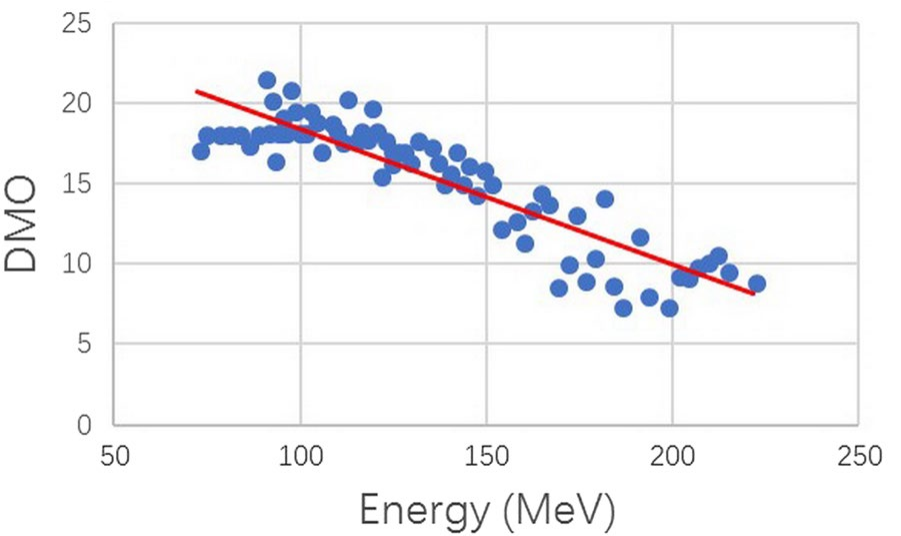
DMO (distance from the point with maximum R^2^ to the origin) plotted against energies for profiles. DMO decreases with increasing energy.

As can be seen from Table 1, different energy ranges had different parameters. Figure 3 shows the power-law exponent plotted against 94 energies and Fig. 4 shows the DMO plotted against the energies. The power-law exponent increased with energy and DMO decreased with increasing energy.

The model was built as described in the Methods. Equation 12 shows our model’s probable prediction for the impact of a proton, including the direct and indirect mechanisms. Equation 15 indicates that once a particle’s number of impacts (*i*) is large enough, further impacts of the particle accelerate growth because there are so many links that can impact particle *A*, showing the characteristics of power law. The power-law exponent is −(1 + 1/λ). The tipping point where *R*_*indirect*_ overtakes *R*_*direct*_ occurs at $i=s=n(\frac{1}{{\rm{\lambda }}}-1)$.

We fit the parameters of λ on the basis of the model and exponent obtained from the statistics. Table 2 shows the fitting parameter values of λ for multiple energy ranges. The data shown in Table 2 indicate that λ increases with energy. Owing to the physical properties of reaction between the proton and matter, the greater the proton energy, the greater the number of indirect impacts and the larger the value of λ, which is consistent with the trend of value obtained from the statistical data and model. In addition, the higher the energy, the larger the power-law exponent (Fig. 3).

**Table 2:** Fitting parameters of λ for multiple energy ranges.

Energy range, MeV	λ
72.5–99.9	0.28 ± 0.05
100–129.9	0.28 ± 0.03
130–159.9	0.29 ± 0.03
160–199.9	0.32 ± 0.05
200–221.8	0.37 ± 0.04

As the energy becomes larger, the λ value becomes larger and the tipping point becomes smaller according to equation 8. The tipping point position obtained from the model was consistent with the trend of DMO obtained from the statistical data (Fig. 4). For the energies between 72.5 and 79.9 MeV, the DMO was about 2.5 cm, whereas for energies between 210 and 221.8 MeV, the DMO was about 0.96 cm. For lower energies (70.9–72.5 MeV), the core and halo distribution overlapped at most distances of interest, and the long-tailed section of the distribution (halo) was not considered important. However, the long tail effect could reach 10 cm even though the DMO occurs at 0.96 cm for high energies (210–221.8 MeV), and it is important to correctly model long-tail effects.

## Methods and Materials

A power-law relationship usually means that some physical properties or probability distributions satisfy the following formula:

1 $${\rm{y}}={{\rm{cx}}}^{{\rm{\alpha }}}$$

The constant α is called the exponent or scaling parameter (slope of the log–log regression line) of the power law.

In the current study, we measured the proton lateral dose distribution (the core and halo) using an ionization chamber in a water tank. In the clinical application of proton therapy, the lateral dose distribution is defined as the “profile.” We used the lateral distance (*r*) in the profile as the independent variable (x) and the lateral dose (*d*) as the dependent variable (y). Because profile data on the positive and negative sides of the x-axis are symmetric, the positive axis is used for testing. The following sections describe the data collection and statistical methods used to establish our model.

### Profile measurement

All profiles were measured at The University of Texas MD Anderson Cancer Center Proton Therapy Center using the spot scanning delivery system, which has been used to treat patients since May 2008^24^. The proton therapy system uses a synchrotron and the Hitachi ProBeat delivery System (Hitachi, Ltd., Tokyo, Japan) with three gantries and two fixed beamlines. The spot scanning beam delivery system offers 94 selectable energies ranging from 72.5 to 221.8 MeV, corresponding to penetrations ranging from 4.0 to 30.6 cm in depth.

We used an ionization chamber to measure the profiles, as described by Sawakuchi *et al*.^25^ and Anand *et al*.^26^. Profiles were measured using a small cylindrical ionization chamber (type 31014, PTW-Freiburg, Freiburg, Germany) in an automated water tank (MP3 Phantom Tank, type L981010, PTW-Freiburg) for all scanning proton energies. The ionization chamber has a sensitive volume with a 0.1-cm radius and 0.5-cm height. The measuring range included extended lateral distances from the central axis at multiple depths, covering low-dose halo regions, which are a factor of 10^4^ lower than the central axis dose.

In the current study, all profile measurements were determined in terms of relative dose. A total of 299 profiles were measured. Two other methods (optically stimulate luminescent detector and Gafchromic EBT film) of measurement were used to verify the reliability of the ionization chamber measurements.

### Regression line fitting

To simplify the process of estimating the relationship, we used log transformation. The x and y variables are log-transformed, so equation (1) can be expressed as:

2 $${\rm{Y}}={\rm{C}}+{\rm{\alpha }}X$$

where Y = log(y), X = log(x), and C = log(c). We can determine whether variables fit to equation (1) by checking whether the log-transformed variables are linearly related. There are two different types of reported power laws: power-law probability distributions^23^ and bivariate power law^27^. For the power-law probability distributions, the regression line fitting method should not be used because regression between x and p(x) (probability density) does not produce a well-defined probability model of x^28^. For the bivariate power law, the least-squares linear regression method is suitable to fit the power-law distribution.

Ordinary least squares (OLS), major axis, and standardized major axis^27^ are the most common methods used to define a line of best fit for a bivariate relationship. The fitting method used for the line differs depending on whether the measurement error is considered. OLS frequently requires the assumption that X is measured without error, whereas the major axis and standardized major axis methods assume that X contains measurement error^29^. The major axis method is also symmetric, meaning that a single line defines the bivariate relationship regardless of which variable is X and which is Y, whereas the OLS method is asymmetric. Because the dose profile data are noise-free in the x-axis and given that the relationship between distance and dose is asymmetric, OLS was used.

In the OLS regression technique, the line is fitted to minimize the sum of squares of residuals from the line. The basic formula is $\sum _{i=1}^{N}{(Y-\bar{Y})}^{2}$, where $\overline{Y}$ is the fitted value of Y. This allows us to test for an association between X and Y. The key statistical parameter is the p value. The strength of the relationship between X and Y also needs to be determined. A suitable statistic to answer this question is the square of the correlation coefficient, R^2^. We used an R package (SMATR) and in-house script to fit the profile data. Details about the use of these statistics and about the underlying formulas and their validation can be found in Warton *et al*.^27^.

In our previous observation, we test all measurement points of the profiles. We found that the power-law relationship applied only to values in the long tail of the profiles. In statistical analysis, we focused on the data in the long-tailed section of the profiles (representing the halo). We applied an algorithm to select the profile data. First, we tested each x as the first value of the dataset to find the maximum R^2^ value and recorded the distance from the point with the maximum R^2^ to the origin. Then, the dataset was made up from the x value with the maximum R^2^ value. Thus, we found the dataset that best matched the power-law relationship in the profiles.

### Model building

We developed a model (focused on the power-law relationship observed in the long-tailed portion of the profiles) aiming to address two points of interest to us. First, we examined proton dose deposition to identify the mechanism behind the power-law phenomenon that occurred in the profiles. Second, we wanted to explain why the power-law parameters for profiles non-universal constants were, varying from one energy to another.

**Figure 5:**
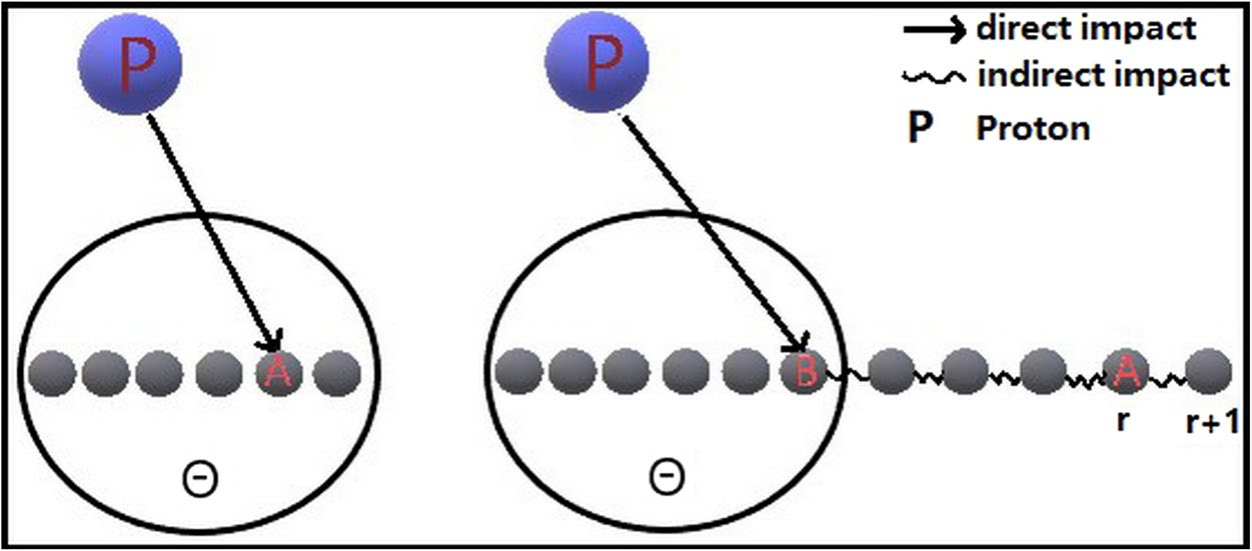
Schematic drawing of the two mechanisms model.

We established a two-mechanism model, comprising the direct mechanism of impact and indirect mechanism of impact. This two-mechanism model is similar to the scientific citation model described in Peterson *et al*.^30^. Figure 5 shows a schematic drawing of the two mechanisms model for Proton depositing dose in the materials. Considering a particle A in the material, if the particle is in the range Θ as shown in the circle, the main interaction of the particle A to the proton is the direct impact. This mechanism is shown at the left panel of the Fig. 5. However, if the particle is outside of the range Θ, the indirect interaction mechanism will be dominant.

As shown in the right panel of the Fig. 5, the proton first impact a particle in the range, the particle B than impact particle A located at position r. As shown by the wiggle line of Fig. 5, the indirect impact from B to A is through a chain reaction. This chain reaction is important for us to understand the power law behavior. If we compare the probability of receiving indirect impacts for the particle in position at r + 1 and r, the particle at position r + 1 (longer distance) will have larger probability of receiving indirect impact than the particle at position r (shorter distance). Once the particle at position r receiving an indirect impact, it will trigger an indirect impact to particle at position r + 1. This means that particle at longer distance will receive more indirect impacts simply because there are more indirect reaction channels. This mechanism is essentially “Richer (longer distance) get richer (more indirect impacts)”.

Assuming that the proton enters the medium (water) from the zero point, the dose is generated by the impact of the proton on particles. At a given time point, a certain number of particles is already at each position on the x-axis. The number of particles is *m*, which is large. Let *n* be the average number of impacts per particle with other particles. Hence the total number of impacts is *mn*. Let *i* be the number of impacts that a particle has received. For example, a particle that has received no impacts from other particles has *i* = 0. Some particles have received many impacts, until the particle energy is converted into dose.

At each time step, an incident proton enters. Now we focus on an particle *A* and discuss the effect of the incident proton on *A*. The probability that this new proton will randomly impact *A* is

3 $${R}_{direct}=1/m$$

Equation 3 is called the direct impact mechanism. Particle *A* can also be subject to an indirect impact mechanism, in which the incident proton impacts other particles, which then impact *A*. Given the physical properties of the reaction between the proton and the particle, any particle having a direct impact on *A* must be within a certain range Θ around *A*. If a particle *A* is outside the range Θ, the particles *B* in Θ are first impacted, then *B* impact *A*. Of course, a particle can pass through multiple impacts before impacting *A*. This process is called the indirect impact mechanism.

Let particle *B* ∈ Θ; in the indirect impact mechanism, the incident proton impacts *B* (either directly or indirectly) and *B* then impacts *A*. If the probability that the new proton impacts *B* is 1 /*m* and the probability that *B* impacts *A* is (1 /*m*) × (1/*n*), then the probability that the incident proton impacts *A* with the indirect impact mechanism is

4 $${R}_{indirect}(i)=(1/m)\times (1/n)\times i$$

Given that this proton has impacted *A*, let λ be the indirect impact probability; the direct impact probability is then 1 − λ. Assuming that *A* has now received *i* impacts, then the number of impacts on particle *A* from an incident proton is I(*i*). I(*i*) can be computed as follows through either the direct or indirect impact mechanism:

5 $${\rm{I}}(i)=n[{\rm{\lambda }}{R}_{indirect}(i)+(1-{\rm{\lambda }}){R}_{direct}]=\frac{n(1-{\rm{\lambda }})}{m}+\frac{i{\rm{\lambda }}}{m}$$

Next, we calculate the impact distribution p(*i*), the fraction of the particles that receive *i* incoming impacts. The number of particles having *i* impacts is *m* × p(*i*) and the number of particles with *i *− 1 impacts is *m* × p(*i *− 1). The number of particles with *i* impacts increases every time a particle with *i *− 1 impacts receives another impact and decreases every time a particle with *i* impacts receives another impact.

When a proton inters,

6 $${\rm{p}}(i)=m[{\rm{I}}(i-1){\rm{p}}(i-1)-{\rm{I}}(i){\rm{p}}(i)]\,\,=[n(1-{\rm{\lambda }})+{\rm{\lambda }}(i-1)]{\rm{p}}(i-1)-[n(1-{\rm{\lambda }})+i{\rm{\lambda }}]{\rm{p}}(i)$$

we get

7 $${\rm{p}}(i)=\frac{s-1+i}{s+1/{\rm{\lambda }}+i}\times p(i-1)$$

where we define

8 $$s=n(\frac{1}{{\rm{\lambda }}}-1)$$

There is no inflow from a lesser bin for p(0). We define 1 as the inflow coming from a incident proton at each time step. So,

9 $${\rm{p}}(0)=1-n(1-{\rm{\lambda }}){\rm{p}}(0),$$

rearranges to

10 $${\rm{p}}(0)=\frac{1}{n-n{\rm{\lambda }}+1}$$

Per equations 7 and 10, we can get

11 $${\rm{p}}(i)=\frac{1}{s{\rm{\lambda }}+1}\times \frac{(s-1+i)!(s+1/{\rm{\lambda }})!}{(s-1)!(s+1/{\rm{\lambda }}+i)!}$$

The general form of the Stirling approximation formula is ${\rm{x}}!\approx {(\frac{{\rm{x}}}{e})}^{{\rm{x}}}$, and we apply it to equation 11, which yields

12 $${\rm{p}}(i)\approx \frac{1}{s{\rm{\lambda }}+1}\times \frac{{(s-1+i)}^{s-1+i}{(s+1/{\rm{\lambda }})}^{s+1/{\rm{\lambda }}}}{{(s-1)}^{s-1}{(s+1/{\rm{\lambda }}+i)}^{s+1/{\rm{\lambda }}+i}}\,\approx \frac{1}{s{\rm{\lambda }}+1}\times \frac{{(s+1/{\rm{\lambda }})}^{s+1/{\rm{\lambda }}}}{{(s-1)}^{s-1}}\times \frac{{(s-1+i)}^{s-1+i}}{{(s+1/{\rm{\lambda }}+i)}^{s+1/{\rm{\lambda }}+i}}\,\approx \frac{1}{s{\rm{\lambda }}+1}\times \frac{{(s+1/{\rm{\lambda }})}^{s+1/{\rm{\lambda }}}}{{(s-1)}^{s-1}}\times {(\frac{s-1+i}{s+1/{\rm{\lambda }}+i})}^{s+i}\times {(s-1+i)}^{-1}\times {(s+1/{\rm{\lambda }}+i)}^{-1/{\rm{\lambda }}}$$

When *i* is large enough (*i*$\gg$s), we have

13 $${(\frac{s-1+i}{s+1/{\rm{\lambda }}+i})}^{s+i}\approx {e}^{-(1+1/{\rm{\lambda }})}$$

and

14 $${(s-1+i)}^{-1}\times {(s+1/{\rm{\lambda }}+i)}^{-1/{\rm{\lambda }}}\approx {i}^{-(1+1/{\rm{\lambda }})}$$

So equation 14 becomes, when *i* is large enough,

15 $${\rm{p}}(i)\approx \frac{1}{s{\rm{\lambda }}+1}\times \frac{{(s+1/{\rm{\lambda }})}^{s+1/{\rm{\lambda }}}}{{(s-1)}^{s-1}}\times {e}^{-(1+1/{\rm{\lambda }})}\times {i}^{-(1+1/{\rm{\lambda }})}$$

Next, we explain the relationship between *i* and distance (*r*), p(*i*), and dose [*d*(*r*)]. Considering that the dose at position r is determined by the number of impacts which particle at position r receives and energy transfer per impacts. In this work, we approximate the same energy will be deposited at position r whenever there is an indirect impact at position r. This is an approximation. We only approximate same energy transfer for the indirect impacts. By using this approximation, we can derive the probability distribution of indirect impacts following the similar method used by Peterson *et al*. Therefore, i and r are proportional and the dose [d(r)] is proportional to p(i).

Thus, *d* can be expressed as the following, with a coefficient (a) according to equation 15:

16 $$d(r)\approx \frac{1}{s{\rm{\lambda }}+1}\times \frac{{(s+1/{\rm{\lambda }})}^{s+1/{\rm{\lambda }}}}{{(s-1)}^{s-1}}\times {e}^{-(1+1/{\rm{\lambda }})}\times {r}^{-(1+1/{\rm{\lambda }})}.$$

### Data availability

Data are fully available through the corresponding author.

## Discussion

The power law has special mathematical properties and can be the result of interesting endogenous processes such as network effects, multiple reactions effects, or feedback loops. A power-law distribution is often considered evidence of the underlying processes constructing the dynamics for these systems^31,32^. Many studies across disciplines have examined the power law, but so far none have used the power law to explain the halo phenomenon in proton dose deposition. In the current study, we used statistical methods to test the measurement data and establish a mechanistic model to describe the power-law relationship between proton lateral dose distribution and the lateral distance, especially at the long tail of the profile. The high correlation coefficient uncovered in our analysis indicates that the relationship between the lateral distance and dose in the halo area follows the power law.

The derivation of our model has some similarities with that of Peterson *et al*., who examined power-law relationships in scientific citations^30^. One key difference is that we applied power law to a natural physical phenomenon. Our model shows that most particles impact at short distances and are converted into dose, but some particles always have more impact and move a longer distance. After the particles move to a certain tipping point, the indirect impact mechanism rapidly increases, causing the particles to move a longer distance to deposit energy, forming the halo.

Our observation that the long-tailed section of a proton dose distribution explained through a simple indirect reaction mechanism, rather than through the first principle, is a novel finding. As we know, the halo consists of charged secondary particles, many but not all of them protons, from elastic interactions with H, elastic and inelastic interactions with O, and non-elastic interactions with O^15^. Our findings suggest that the net effects of these complex physical reactions lead to a very simple power-law distribution. The power-law interpretation of physics data can also bring some insight of the social phenomena such as scientific paper citation. One interesting result in this work is that high-energy proton will have a longer tail compared to low-energy proton. The “high-energy proton” in the current study is analogous to Peterson *et al*.’s^30^ “highly cited group” of scientific citations, which also exhibited a longer tail than the group with a lower H-index.

**Figure 6:**
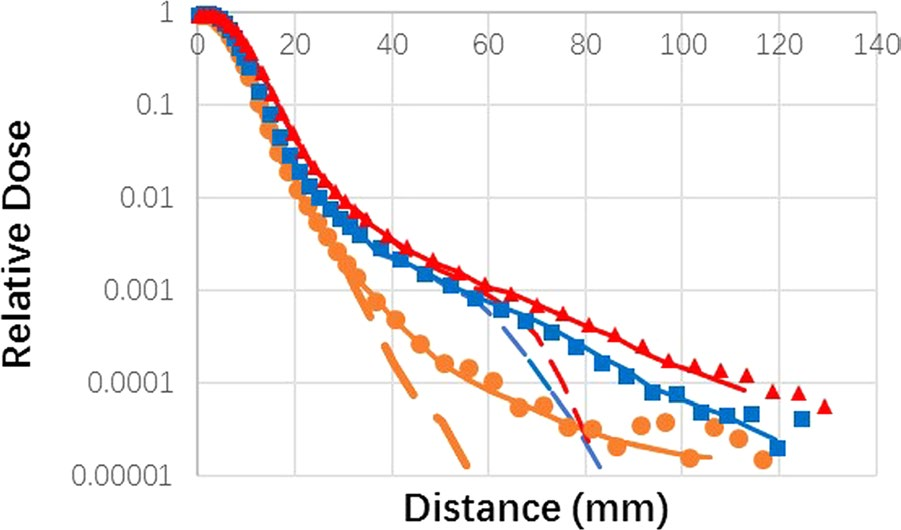
The lateral profiles of individual scanning spots modeled at various depths using the double-Gaussian functions and power-law function (solid lines) and double-Gaussian functions only (dashed lines). The measured lateral profiles of 221.8 MeV at different depths are indicated by the brown circle (depths: 2 cm), blue square (depths: 10 cm) and red triangle (depths: 20 cm) markers, respectively.

In all proton dose calculation models, the functions describing the off-axis lateral distribution are Gaussian. Some studies have used one Gaussian and some studies have used two or more to handle large-angle scatters^9^. For example, the pencil beam distribution was modeled using three Gaussian functions in our treatment planning system (Eclipse 8.9; Varian Medical Systems, Alta Palo, CA)^18^. During the clinical commissioning of the scanning beam, we previously observed that the measured dose of the treatment planning system at multiple depths in the square field cannot be accurately predicted as a function of field size. The difference was found at the medium depth of the high-energy beam^33^. In fact, despite the use of multiple Gaussian functions, or the addition of various correction factors, the halo phenomenon was not described very well. The power-law relationship may provide a novel and accurate alternative model of proton dose distribution. Figure 6 show that low-dose halos in single-spot profiles of 221.8 MeV could be adequately modeled with the addition of the power-law function to a double-Gaussian function. This figure shows the lateral profiles of individual scanning spots modeled at various depths using the double-Gaussian functions and power-law function (solid lines) and double-Gaussian functions only (dashed lines). The measured lateral profiles at different depths are indicated by the brown circle (depths: 2 cm), blue square (depths: 10 cm) and red triangle (depths: 20 cm) markers, respectively. The lateral profiles modeled with the two Gaussian components and power-law component showed better agreement with the measured results compared with the model using the two Gaussian components only. However, this model with power-law function needs further study.
